# Ishiguro technique percutaneous metallic bone needle closed reduction for Judet IV radial head fractures in children: A case report (CARE-compliant)

**DOI:** 10.1097/MD.0000000000043680

**Published:** 2025-08-01

**Authors:** Wenke Chen, Yalong Ma, Lin Feng, Chongfang Zhang, Jinsong Sun, Nuan Han

**Affiliations:** a Department of Pediatric Surgery, Affiliated Hospital of Jining Medical University, Jining, Shandong Province, China; b Clinical Medical College of Jining Medical University (Affiliated Hospital), Jining, Shandong Province, China.

**Keywords:** case report, child, Ishiguro technique, radial head fractures

## Abstract

**Rationale:**

Fractures of the radial head in children constitute 5% to 17% of all elbow fractures and 1% of all pediatric fractures. As intra-articular injuries they pose significant challenges due to the high risk of loose body formation and ischemic necrosis, which can lead to long-term joint dysfunction and reduced quality of life. Although advances have been made in pediatric orthopedic surgery, developing minimally invasive and effective treatment strategies remains a crucial research focus for optimizing functional recovery and reducing complications.

**Patient concerns:**

An 8-year-old female patient sustained a left upper limb injury during a school recreational activity, resulting in immediate pain and restricted movement. Physical examination revealed mild swelling and tenderness in the left elbow, with no obvious deformity, but limited range of motion in the elbow joint and forearm. Radial artery pulsations were palpable, and finger movement and sensation were normal, indicating intact neurovascular function.

**Diagnosis:**

Fracture of the left radial head.

**Interventions:**

Using the Ishiguro technique with two 1.5-cm metallic bone pins, stable reduction of the fracture fragments was achieved. After trimming the pin ends, they were left outside the skin for fixation. The elbow joint was immobilized at approximately 80° of flexion using a plaster splint. The patient remained hemodynamically stable during surgery and was stable postoperatively when transferred to the ward.

**Outcomes:**

After 6 weeks of plaster immobilization, the metallic bone pins were removed, revealing good fracture healing.

**Lesson:**

This case report demonstrates the successful application of the Ishiguro technique, traditionally used for treating swan-neck deformities caused by avulsion fractures of the distal phalanx base, in the closed reduction and stable internal fixation of a pediatric Judet IV radial head fracture. By repurposing this technique, we have expanded its clinical utility and provided a minimally invasive treatment option for complex pediatric radial head fractures. This approach may enhance functional recovery, reduce surgical trauma, and offer potential cost-effectiveness, holding positive implications for advancing treatment strategies in pediatric orthopedics.

## 1. Introduction

Radial head fractures account for a significant proportion (5–17%) of elbow fractures in children and approximately 1% of all pediatric fractures. The epiphyseal center of the proximal radius manifests as a small, round ossification center by the age of 5, with occasional bifurcation that may mimic a fracture. By 7 years, a typical ossification nucleus is evident. Fusion of the ossification center with the main radius occurs between the ages of 16 and 18. The radial head is entirely encapsulated within the elbow joint, devoid of surrounding ligaments or tendons. Blood supply to the radial head is solely reliant on vessels within the synovium surrounding the radial neck until epiphyseal closure. As radial head fractures are intra-articular, they may lead to loose bodies and are prone to avascular necrosis.^[1]^ The Ishiguro technique, initially utilized for mallet finger deformities,^[2]^ demonstrates promise in facilitating closed reduction and stable internal fixation of radial head fractures. Herein, we document this infrequently encountered injury and surgical technique, aiming to contribute to clinical decision-making in this area.

## 2. Case presentation

**Figure 1.**
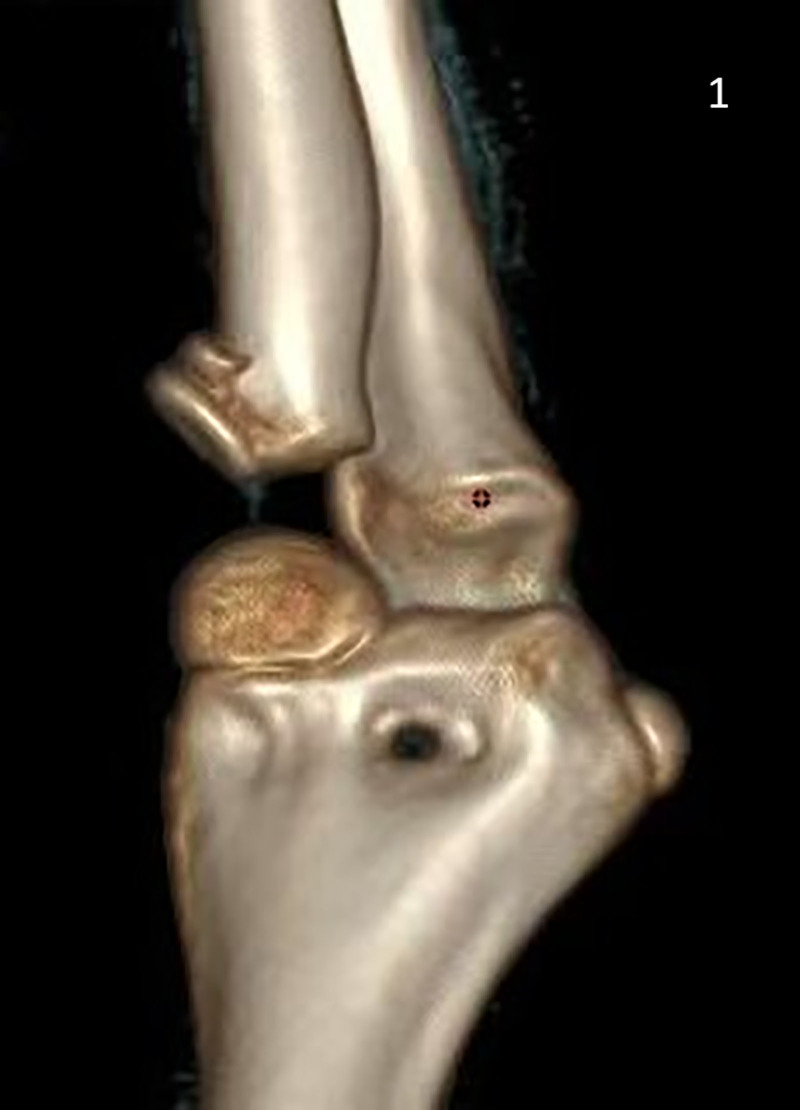
3D CT examination suggested “left radial head fracture.” CT = computed tomography.

An 8-year-old female patient sustained an injury to her left upper limb during recreational activities at school. Immediate pain and immobilization of the affected limb ensued. Physical examination revealed mild swelling, tenderness at the left elbow, nonapparent deformity, and limited range of motion in the elbow and forearm. The radial pulse was palpable, indicating adequate blood flow to the extremities. Full range of motion and normal sensation were noted in the fingers of the left hand. A 3-dimensional computed tomography scan upon admission confirmed the diagnosis of a left radial head fracture (Fig. 1). Based on the fracture’s angulation and displacement characteristics, it was classified as a Judet IV-type radial head fracture.

**Figure 2.**
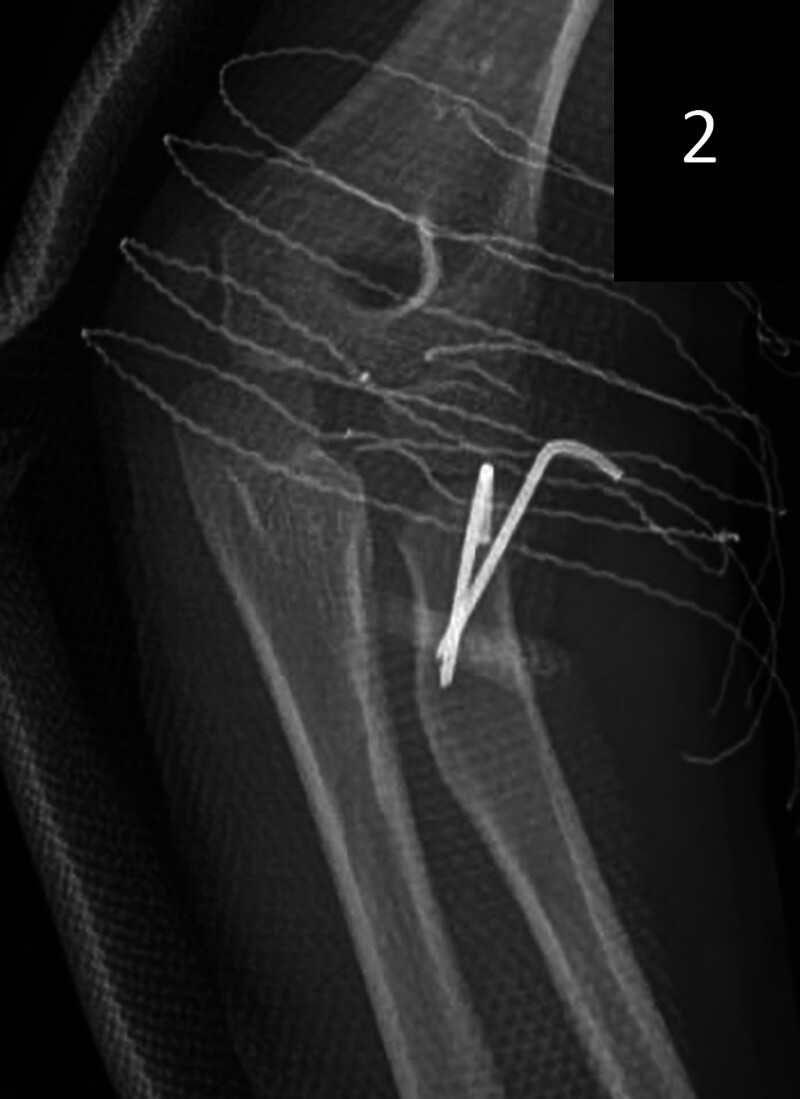
Immobilization of the elbow in the functional position with plaster support at approximately 80° of flexion.

Surgery was promptly performed upon admission. The patient’s identity and surgical plan were verified by the circulating nurse and anesthesiologist. General anesthesia, augmented by a nerve block, was administered. In the supine position, routine disinfection and draping protocols were followed. Intraoperative C-arm fluoroscopy visualized a left radial head fracture with lateral and inferior displacement. Manual reduction attempts were made; however, C-arm fluoroscopy confirmed suboptimal reduction, with residual mild lateral and inferior displacement of the fracture fragment. Consequently, two 1.5-cm metal bone pins, assisted by the Ishiguro technique, were utilized to achieve stable fracture fragment positioning. The reduction was deemed satisfactory, with the pin tails trimmed and left external to the skin, followed by dressing for fixation. The elbow was immobilized in a functional position at approximately 80° with a plaster splint (Fig. 2). The patient’s vital signs remained stable throughout the procedure, and she was subsequently transferred to the ward in stable condition. The Medical Research Ethics Committee of Affiliated Hospital of Jining Medical University approved the study.

## 3. Outcomes

**Figure 3.**
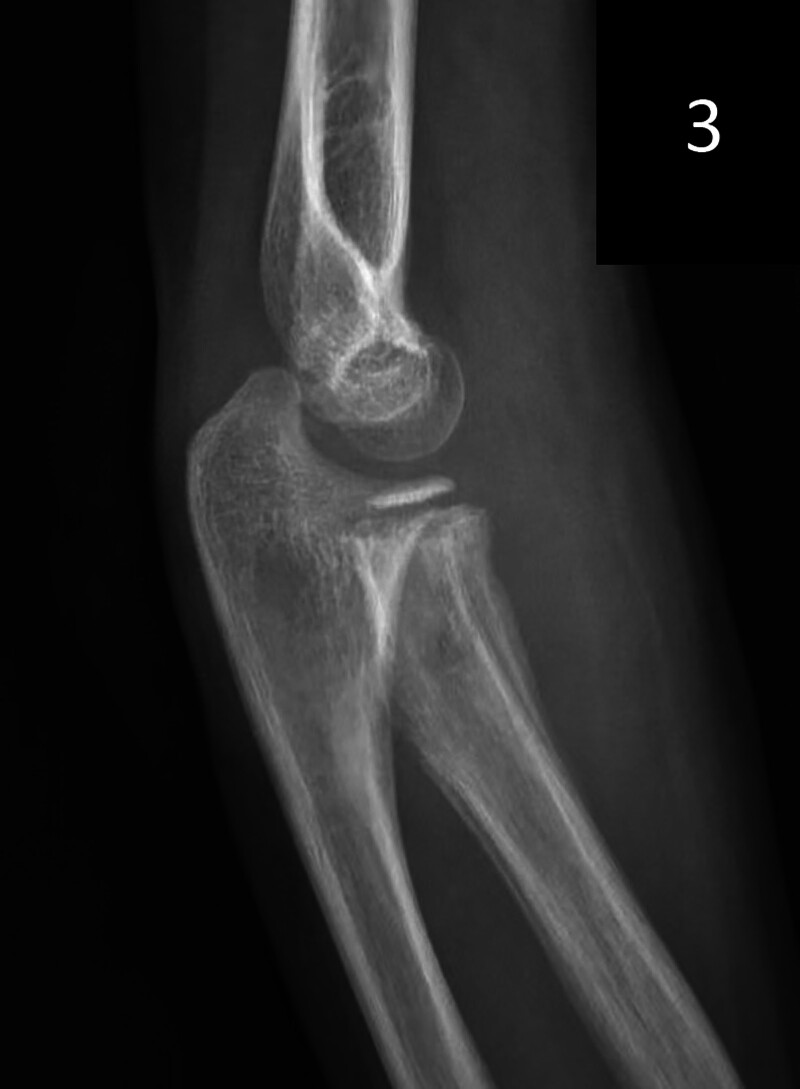
The elbow was fixed with plaster for 6 weeks, the metal bone pins were removed, and the bone healed well.

**Figure 4.**
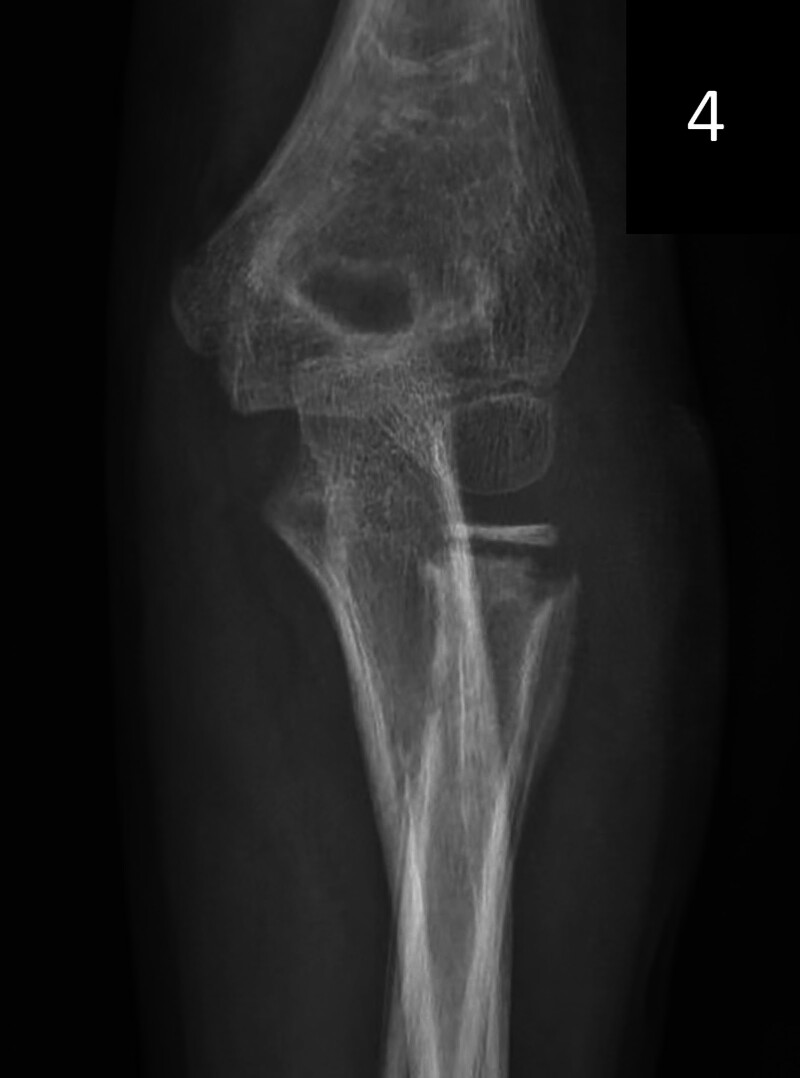
The elbow was fixed with plaster for 6 weeks, the metal bone pins were removed, and the bone healed well.

The case underwent Ishiguro technique-assisted closed reduction successfully, avoiding open surgery. The skin incision was small and required no suturing, just a sterile dressing. Postoperative pain was mild, needing no extra analgesics. The patient resumed liquid intake 6 hours post-surgery and a normal diet the next day. Hospitalization lasted 3 days with no complications like infection or nerve injury. At 6-week follow-up, the fracture healed well with normal joint function and no recurrence or late displacement (Figs. 3 and 4). After metal-pin removal, there was minimal scarring at the elbow, only tiny pin-prick marks, achieving a good cosmetic result.

## 4. Discussion

Radial head fractures in children are frequently associated with distal humerus fractures, olecranon fractures, and radioulnar dislocations.^[3,4]^ These fractures typically affect children aged 4 to 14, coinciding with the onset of radial head ossification at age 5 and epiphyseal closure occurring later in adolescence. Due to the unique anatomical and developmental characteristics of the radial neck in children, there is no literature on the optimal treatment strategy. The most prevalent mechanism of injury involves a direct fall, often resulting from indirect trauma, characterized by a fall onto an outstretched, pronated forearm with elbow extension. This leads to mechanical force transmission, impacting the radial head and capitulum of the humerus, potentially causing radial neck fractures or separation of the radial head epiphysis. Fracture fragments are often displaced inferiorly or laterally and inferiorly.^[5]^ The biceps and supinator muscles pull the distal fracture fragment toward the ulnar side; however, complete displacement is uncommon. In our case, significant fracture displacement increased the complexity of manual reduction.

The consensus among scholars dictates that an acceptable range of angulation for pediatric radial neck fractures during treatment is <30°, with displacement <2 mm. Children’s bones have a remarkable capacity for self-modeling and fracture remodeling, particularly at younger ages. Angulation exceeding 60° is unacceptable regardless of age. Open reduction surgery is indicated when closed reduction fails, most commonly seen in irreducible radial head fractures. For significantly displaced Judet IV-type fractures, closed reduction methods should be attempted initially, with open reduction considered only after the failure of closed reduction and less invasive techniques. The lateral posterior (Kocher) approach is the most frequently utilized surgical approach. If instability persists postreduction, internal fixation using Kirschner wires or flexible intramedullary nails is recommended.^[6]^

The Ishiguro technique necessitates 2 metal bone pins: one to stabilize the avulsed fracture fragment and another for fracture fixation.^[7]^ With the elbow extended, a 1.5 cm metal bone pin is inserted from the radial side proximal to the laterally and inferiorly displaced radial head fracture fragment, pressing it ulnarward to reduce the fragment. Once near fracture apposition is restored, a second 1.5 cm metal bone pin is inserted through the lateral elbow across the radial head fracture to maintain apposition. C-arm confirmation of satisfactory fracture reduction ensues, with the elbow immobilized in a functional position at 80° with a plaster splint to prevent reduction loss and joint contracture. In this case, the Ishiguro technique successfully treated a child with a Judet IV-type radial head fracture, utilizing the metal bone pins’ blocking effect to facilitate the reduction of completely displaced fracture fragments. This avoided the complications associated with open reduction, including blood supply damage, nerve injury, joint capsule trauma, and significant surgical trauma. The patient exhibited satisfactory fracture healing and functional recovery of the affected limb.

## 5. Conclusion

We report a case of a child with a Judet IV-type radial head fracture successfully treated using closed reduction with the Ishiguro technique, yielding favorable reduction outcomes. Considering the potential adverse functional outcomes associated with open surgery, closed reduction techniques are preferred when feasible. The Ishiguro technique emerges as a viable option for closed reduction treatment of radial head fractures in children. However, this study has limitations. It only analyzed one case of pediatric Judet IV radial head fracture. The small sample size restricts the generalizability and reliability of the results. Also, the Ishiguro technique, a minimally invasive but technically demanding method, relies heavily on the operator’s skill and experience for successful outcomes. To enhance the generalizability and reliability of the conclusions, future studies should increase the sample size and ensure its diversity and representativeness.

## Author contributions

**Conceptualization:** Wenke Chen, Lin Feng, Nuan Han.

**Data curation:** Wenke Chen, Yalong Ma, Lin Feng, Nuan Han.

**Investigation:** Yalong Ma, Chongfang Zhang.

**Methodology:** Yalong Ma.

**Validation:** Lin Feng, Chongfang Zhang, Jinsong Sun.

**Visualization:** Chongfang Zhang, Jinsong Sun.

**Writing – original draft:** Wenke Chen.

**Writing – review & editing:** Nuan Han.
